# (*E*)-*N*-[(2-Eth­oxy­naphthalen-1-yl)methyl­idene]-2-ethyl­aniline

**DOI:** 10.1107/S1600536812043097

**Published:** 2012-10-20

**Authors:** Hakan Kargılı, Mustafa Macit, Gökhan Alpaslan, Canan Kazak, Ahmet Erdönmez

**Affiliations:** a Department of Physics, Faculty of Arts & Science, Ondokuz Mayıs University, TR-55139 Kurupelit-Samsun, Turkey; b Department of Chemistry, Faculty of Arts & Science, Ondokuz Mayıs University, TR-55139 Kurupelit-Samsun, Turkey; c Department of Medical Services, and Techniques, Vocational School of Health Services, Giresun University, TR-28200 Giresun, Turkey

## Abstract

In the title compound, C_21_H_21_NO, the dihedral angle between the naphthalene ring system and the benzene ring is 64.61 (6)°. The mol­ecular structure is stabilized by an intra­molecular C—H⋯N hydrogen bond.

## Related literature

For biological properties of Schiff bases, see: Lozier *et al.* (1975 ▶). For the coordination chemistry of Schiff bases, see: Kargar *et al.* (2009 ▶); Yeap *et al.* (2009 ▶). For hydrogen-bonding motifs, see: Bernstein *et al.* (1995 ▶). For a related structure, see: Vesek *et al.* (2012 ▶).
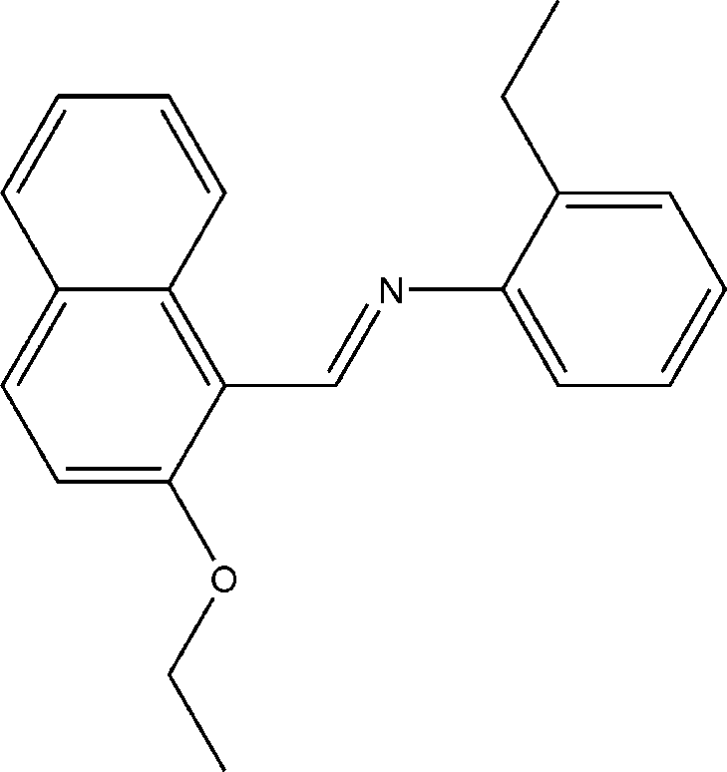


## Experimental

### 

#### Crystal data

C_21_H_21_NO
*M* *_r_* = 303.39
Monoclinic, 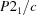

*a* = 11.6011 (11) Å
*b* = 20.457 (3) Å
*c* = 7.4335 (7) Å
β = 101.303 (8)°
*V* = 1730.0 (3) Å^3^
*Z* = 4
Mo *K*α radiation
μ = 0.07 mm^−1^
*T* = 296 K
0.71 × 0.55 × 0.36 mm

#### Data collection

Stoe IPDS-II diffractometer
14212 measured reflections
3403 independent reflections
1769 reflections with *I* > 2σ(*I*)
*R*_int_ = 0.055

#### Refinement

*R*[*F* ^2^ > 2σ(*F* ^2^)] = 0.065
*wR*(*F* ^2^) = 0.211
*S* = 0.94
3403 reflections
209 parameters
16 restraints
H-atom parameters constrained
Δρ_max_ = 0.45 e Å^−3^
Δρ_min_ = −0.31 e Å^−3^

### 

Data collection: *X-AREA* (Stoe & Cie, 2002 ▶); cell refinement: *X-AREA*; data reduction: *X-RED32* (Stoe & Cie, 2002 ▶); program(s) used to solve structure: *SHELXS97* (Sheldrick, 2008 ▶); program(s) used to refine structure: *SHELXL97* (Sheldrick, 2008 ▶); molecular graphics: *ORTEP-3 for Windows* (Farrugia, 2012 ▶); software used to prepare material for publication: *WinGX* (Farrugia, 2012 ▶).

## Supplementary Material

Crystal structure: contains datablock(s) I, global. DOI: 10.1107/S1600536812043097/fj2600sup1.cif

Structure factors: contains datablock(s) I. DOI: 10.1107/S1600536812043097/fj2600Isup2.hkl

Supplementary material file. DOI: 10.1107/S1600536812043097/fj2600Isup3.cml

Additional supplementary materials:  crystallographic information; 3D view; checkCIF report

## Figures and Tables

**Table 1:** Hydrogen-bond geometry (Å, °)

*D*—H⋯*A*	*D*—H	H⋯*A*	*D*⋯*A*	*D*—H⋯*A*
C9—H9⋯N1	0.93	2.32	2.961 (4)	126

**Fig. 1.**
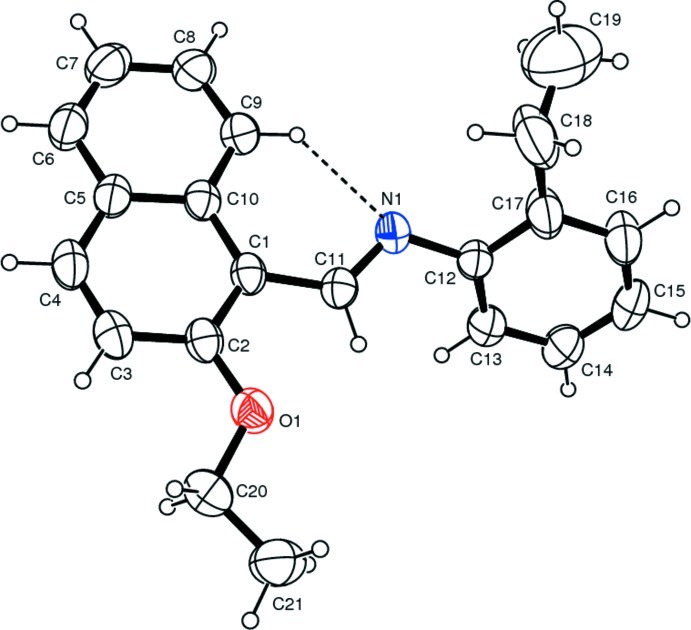
The molecular structure of (I), showing the atom-numbering scheme. Displacement ellipsoids are drawn at the 30% probability.

## supplementary crystallographic information

### Comment

Schiff bases often exhibit various biological activities and in many cases were shown to have antibacterial, anticancer, anti-inflammatory and antitoxic properties (Lozier *et al.*, 1975). Schiff bases have also been used as versatile ligands in coordination chemistry (Kargar *et al.*, 2009; Yeap *et al.*, 2009). In this paper, the structure of the title compound, is reported. An ORTEP-3 (Farrugia, 2012) plot of the molecule of (I) is shown in Fig.1. The geometric parameters in (I) are comparable with those in (E)-3-Chloro-N-[(2-ethoxynaphthalen -1-yl)methylidene]aniline (Vesek *et al.*, 2012). The dihedral angle between the naphthalene ring and the benzene ring is 64.61 (6)°. The molecular structure is stabilized by a C9-H9···N1 intramolecular hydrogen bond which generates an S(6) ring motif (Bernstein *et al.*, 1995).

### Experimental

The compound (E)-N-((2-ethoxynaphthalen-1-yl)methylene)-2-ethylaniline was prepared by refluxing a mixture of a solution containing 2-ethoxy-1-naphthaldehyde (20,0 mg, 0,1 mmol) in ethanol (20 ml) and a solution containing 2-ethylaniline (12,12 mg, 0,1 mmol) in ethanol (20 ml). The reaction mixture was stirred for 5 hour under reflux. Single crystals of the title compound for x-ray analysis were obtained by slow evaporation of an ethanol solution (Yield 62%; m.p.376-378 K).

### Refinement

All H atoms bound to C atoms were refined using a riding model, with C-H = 0.93 Å and *U*_iso_(H) = 1.2 *U*_eq_(C) for aromatic C atoms, C-H=0.97 Å and *U*_iso_(H) = 1.2 *U*_eq_(C) for methylene C atoms, and C-H=0.96 Å and *U*_iso_(H) = 1.5 *U*_eq_(C) for methyl C atoms. Restraints and constraints (ISOR, DFIX, DELU) were used in order to maintain a reasonable geometry and atomic displacement parameters for C17, C18 and C19 atoms.

### Crystal data

**Table:**

C_21_H_21_NO	*F*(000) = 648
*M**_r_* = 303.39	*D*_x_ = 1.165 Mg m^−^^3^
Monoclinic, *P*2_1_/*c*	Mo *K*α radiation, λ = 0.71073 Å
Hall symbol: -P 2ybc	Cell parameters from 10574 reflections
*a* = 11.6011 (11) Å	θ = 1.8–27.9°
*b* = 20.457 (3) Å	µ = 0.07 mm^−^^1^
*c* = 7.4335 (7) Å	*T* = 296 K
β = 101.303 (8)°	Prism, yellow
*V* = 1730.0 (3) Å^3^	0.71 × 0.55 × 0.36 mm
*Z* = 4	

### Data collection

**Table:**

Stoe IPDS-II diffractometer	1769 reflections with *I* > 2σ(*I*)
Radiation source: fine-focus sealed tube	*R*_int_ = 0.055
Graphite monochromator	θ_max_ = 26.0°, θ_min_ = 1.8°
Detector resolution: 6.67 pixels mm^-1^	*h* = −14→14
ω scans	*k* = −25→25
14212 measured reflections	*l* = −9→9
3403 independent reflections	

### Refinement

**Table:**

Refinement on *F*^2^	Primary atom site location: structure-invariant direct methods
Least-squares matrix: full	Secondary atom site location: difference Fourier map
*R*[*F*^2^ > 2σ(*F*^2^)] = 0.065	Hydrogen site location: inferred from neighbouring sites
*wR*(*F*^2^) = 0.211	H-atom parameters constrained
*S* = 0.94	*w* = 1/[σ^2^(*F*_o_^2^) + (0.1288*P*)^2^] where *P* = (*F*_o_^2^ + 2*F*_c_^2^)/3
3403 reflections	(Δ/σ)_max_ < 0.001
209 parameters	Δρ_max_ = 0.45 e Å^−^^3^
16 restraints	Δρ_min_ = −0.31 e Å^−^^3^

### Special details

**Table:**

Geometry. All esds (except the esd in the dihedral angle between two l.s. planes) are estimated using the full covariance matrix. The cell esds are taken into account individually in the estimation of esds in distances, angles and torsion angles; correlations between esds in cell parameters are only used when they are defined by crystal symmetry. An approximate (isotropic) treatment of cell esds is used for estimating esds involving l.s. planes.
Refinement. Refinement of F^2^ against ALL reflections. The weighted R-factor wR and goodness of fit S are based on F^2^, conventional R-factors R are based on F, with F set to zero for negative F^2^. The threshold expression of F^2^ > 2sigma(F^2^) is used only for calculating R-factors(gt) etc. and is not relevant to the choice of reflections for refinement. R-factors based on F^2^ are statistically about twice as large as those based on F, and R- factors based on ALL data will be even larger.

### Fractional atomic coordinates and isotropic or equivalent isotropic displacement parameters (Å^2^)

**Table:**

	*x*	*y*	*z*	*U*_iso_*/*U*_eq_	
C1	0.4025 (2)	0.19226 (13)	0.3272 (3)	0.0619 (7)	
C2	0.3508 (2)	0.25280 (13)	0.2813 (4)	0.0664 (7)	
C3	0.2305 (3)	0.25814 (17)	0.2024 (4)	0.0784 (8)	
H3	0.1976	0.2989	0.1686	0.094*	
C4	0.1635 (3)	0.20410 (18)	0.1760 (4)	0.0810 (9)	
H4	0.0837	0.2085	0.1273	0.097*	
C5	0.2097 (2)	0.14074 (15)	0.2197 (4)	0.0707 (8)	
C6	0.1392 (3)	0.08513 (19)	0.1902 (5)	0.0905 (10)	
H6	0.0597	0.0897	0.1395	0.109*	
C7	0.1834 (3)	0.0246 (2)	0.2336 (5)	0.1001 (11)	
H7	0.1349	−0.0120	0.2132	0.120*	
C8	0.3027 (3)	0.01776 (16)	0.3093 (5)	0.0901 (9)	
H8	0.3332	−0.0237	0.3402	0.108*	
C9	0.3751 (3)	0.07046 (14)	0.3386 (4)	0.0753 (8)	
H9	0.4544	0.0645	0.3880	0.090*	
C10	0.3315 (2)	0.13437 (13)	0.2950 (4)	0.0620 (7)	
C11	0.5272 (2)	0.19292 (13)	0.4136 (4)	0.0642 (7)	
H11	0.5578	0.2324	0.4633	0.077*	
C12	0.7140 (2)	0.15485 (13)	0.5261 (4)	0.0661 (7)	
C13	0.7377 (3)	0.18615 (15)	0.6913 (4)	0.0808 (9)	
H13	0.6761	0.2040	0.7386	0.097*	
C14	0.8499 (3)	0.19186 (17)	0.7886 (5)	0.0958 (11)	
H14	0.8640	0.2131	0.9015	0.115*	
C15	0.9404 (3)	0.16685 (18)	0.7216 (5)	0.0938 (10)	
H15	1.0170	0.1712	0.7871	0.113*	
C16	0.9191 (3)	0.13519 (19)	0.5575 (5)	0.1005 (12)	
H16	0.9819	0.1179	0.5124	0.121*	
C17	0.8060 (3)	0.12796 (18)	0.4555 (5)	0.0971 (10)	
C18	0.7839 (4)	0.0973 (4)	0.2575 (7)	0.184 (3)	
H18A	0.7015	0.1018	0.2010	0.221*	
H18B	0.8298	0.1208	0.1825	0.221*	
C19	0.8162 (8)	0.0289 (3)	0.2652 (10)	0.226 (3)	
H19A	0.8030	0.0113	0.1431	0.339*	
H19B	0.7692	0.0055	0.3366	0.339*	
H19C	0.8978	0.0245	0.3209	0.339*	
C20	0.3711 (3)	0.36953 (14)	0.3110 (4)	0.0817 (9)	
H20A	0.3133	0.3707	0.3892	0.098*	
H20B	0.3326	0.3812	0.1873	0.098*	
C21	0.4679 (4)	0.41565 (17)	0.3792 (6)	0.1107 (12)	
H21A	0.4369	0.4591	0.3800	0.166*	
H21B	0.5243	0.4142	0.3004	0.166*	
H21C	0.5054	0.4036	0.5015	0.166*	
N1	0.59740 (19)	0.14530 (11)	0.4274 (3)	0.0729 (7)	
O1	0.42183 (17)	0.30550 (9)	0.3129 (3)	0.0820 (6)	

### Atomic displacement parameters (Å^2^)

**Table:**

	*U*^11^	*U*^22^	*U*^33^	*U*^12^	*U*^13^	*U*^23^
C1	0.0527 (14)	0.0783 (18)	0.0563 (15)	0.0040 (12)	0.0148 (11)	0.0010 (12)
C2	0.0592 (15)	0.0801 (18)	0.0615 (16)	0.0058 (13)	0.0162 (12)	0.0046 (13)
C3	0.0663 (18)	0.095 (2)	0.0730 (19)	0.0177 (15)	0.0104 (14)	0.0069 (15)
C4	0.0559 (16)	0.112 (2)	0.071 (2)	0.0119 (16)	0.0036 (14)	−0.0018 (17)
C5	0.0571 (16)	0.096 (2)	0.0588 (17)	−0.0025 (14)	0.0121 (12)	−0.0122 (14)
C6	0.0628 (18)	0.116 (3)	0.091 (2)	−0.0098 (18)	0.0125 (15)	−0.018 (2)
C7	0.088 (2)	0.104 (3)	0.112 (3)	−0.029 (2)	0.029 (2)	−0.025 (2)
C8	0.084 (2)	0.080 (2)	0.111 (3)	−0.0078 (17)	0.0304 (18)	−0.0116 (18)
C9	0.0662 (17)	0.0798 (19)	0.082 (2)	0.0005 (15)	0.0195 (14)	−0.0035 (15)
C10	0.0566 (15)	0.0787 (18)	0.0532 (15)	0.0015 (12)	0.0166 (11)	−0.0037 (12)
C11	0.0578 (15)	0.0722 (16)	0.0633 (16)	−0.0008 (12)	0.0133 (12)	0.0009 (12)
C12	0.0543 (14)	0.0678 (16)	0.0740 (19)	−0.0001 (12)	0.0075 (12)	0.0007 (13)
C13	0.0674 (17)	0.088 (2)	0.084 (2)	0.0005 (14)	0.0091 (15)	−0.0120 (16)
C14	0.077 (2)	0.109 (3)	0.094 (2)	−0.0061 (18)	−0.0013 (18)	−0.0219 (19)
C15	0.0596 (18)	0.112 (3)	0.101 (3)	−0.0172 (17)	−0.0050 (17)	0.009 (2)
C16	0.0576 (18)	0.145 (3)	0.097 (3)	0.0103 (18)	0.0111 (17)	−0.006 (2)
C17	0.0582 (18)	0.129 (3)	0.101 (2)	0.0164 (18)	0.0087 (16)	−0.0085 (19)
C18	0.085 (3)	0.291 (5)	0.168 (4)	0.056 (4)	0.005 (3)	−0.116 (4)
C19	0.272 (7)	0.219 (5)	0.197 (6)	−0.055 (5)	0.071 (5)	−0.055 (5)
C20	0.097 (2)	0.079 (2)	0.0715 (19)	0.0159 (17)	0.0227 (16)	0.0117 (15)
C21	0.125 (3)	0.084 (2)	0.124 (3)	−0.006 (2)	0.027 (2)	−0.006 (2)
N1	0.0529 (12)	0.0801 (15)	0.0832 (17)	0.0061 (11)	0.0072 (11)	−0.0080 (12)
O1	0.0715 (12)	0.0713 (13)	0.1033 (16)	0.0058 (10)	0.0174 (11)	0.0105 (10)

### Geometric parameters (Å, º)

**Table:**

C1—C2	1.389 (4)	C12—N1	1.420 (3)
C1—C10	1.435 (4)	C13—C14	1.365 (4)
C1—C11	1.464 (3)	C13—H13	0.9300
C2—O1	1.349 (3)	C14—C15	1.348 (5)
C2—C3	1.408 (4)	C14—H14	0.9300
C3—C4	1.344 (4)	C15—C16	1.360 (5)
C3—H3	0.9300	C15—H15	0.9300
C4—C5	1.416 (4)	C16—C17	1.388 (4)
C4—H4	0.9300	C16—H16	0.9300
C5—C6	1.393 (4)	C17—C18	1.573 (5)
C5—C10	1.420 (4)	C18—C19	1.449 (7)
C6—C7	1.356 (5)	C18—H18A	0.9700
C6—H6	0.9300	C18—H18B	0.9700
C7—C8	1.395 (5)	C19—H19A	0.9600
C7—H7	0.9300	C19—H19B	0.9600
C8—C9	1.357 (4)	C19—H19C	0.9600
C8—H8	0.9300	C20—O1	1.435 (3)
C9—C10	1.416 (4)	C20—C21	1.478 (5)
C9—H9	0.9300	C20—H20A	0.9700
C11—N1	1.261 (3)	C20—H20B	0.9700
C11—H11	0.9300	C21—H21A	0.9600
C12—C13	1.364 (4)	C21—H21B	0.9600
C12—C17	1.392 (4)	C21—H21C	0.9600
			
C2—C1—C10	119.3 (2)	C15—C14—C13	120.2 (3)
C2—C1—C11	116.2 (2)	C15—C14—H14	119.9
C10—C1—C11	124.5 (2)	C13—C14—H14	119.9
O1—C2—C1	116.7 (2)	C14—C15—C16	119.6 (3)
O1—C2—C3	122.2 (3)	C14—C15—H15	120.2
C1—C2—C3	121.1 (3)	C16—C15—H15	120.2
C4—C3—C2	119.7 (3)	C15—C16—C17	121.8 (3)
C4—C3—H3	120.2	C15—C16—H16	119.1
C2—C3—H3	120.2	C17—C16—H16	119.1
C3—C4—C5	122.5 (3)	C16—C17—C12	117.7 (3)
C3—C4—H4	118.7	C16—C17—C18	121.1 (3)
C5—C4—H4	118.7	C12—C17—C18	120.9 (3)
C6—C5—C4	121.8 (3)	C19—C18—C17	110.8 (5)
C6—C5—C10	119.7 (3)	C19—C18—H18A	109.5
C4—C5—C10	118.5 (3)	C17—C18—H18A	109.5
C7—C6—C5	121.7 (3)	C19—C18—H18B	109.5
C7—C6—H6	119.1	C17—C18—H18B	109.5
C5—C6—H6	119.1	H18A—C18—H18B	108.1
C6—C7—C8	119.1 (3)	C18—C19—H19A	109.5
C6—C7—H7	120.4	C18—C19—H19B	109.5
C8—C7—H7	120.4	H19A—C19—H19B	109.5
C9—C8—C7	121.3 (3)	C18—C19—H19C	109.5
C9—C8—H8	119.4	H19A—C19—H19C	109.5
C7—C8—H8	119.4	H19B—C19—H19C	109.5
C8—C9—C10	121.0 (3)	O1—C20—C21	107.3 (3)
C8—C9—H9	119.5	O1—C20—H20A	110.3
C10—C9—H9	119.5	C21—C20—H20A	110.3
C9—C10—C5	117.2 (2)	O1—C20—H20B	110.3
C9—C10—C1	123.8 (2)	C21—C20—H20B	110.3
C5—C10—C1	118.9 (2)	H20A—C20—H20B	108.5
N1—C11—C1	126.6 (3)	C20—C21—H21A	109.5
N1—C11—H11	116.7	C20—C21—H21B	109.5
C1—C11—H11	116.7	H21A—C21—H21B	109.5
C13—C12—C17	119.4 (3)	C20—C21—H21C	109.5
C13—C12—N1	122.2 (3)	H21A—C21—H21C	109.5
C17—C12—N1	118.3 (3)	H21B—C21—H21C	109.5
C12—C13—C14	121.4 (3)	C11—N1—C12	118.0 (2)
C12—C13—H13	119.3	C2—O1—C20	119.5 (2)
C14—C13—H13	119.3		
			
C10—C1—C2—O1	−179.4 (2)	C11—C1—C10—C5	176.3 (2)
C11—C1—C2—O1	3.0 (4)	C2—C1—C11—N1	−162.3 (3)
C10—C1—C2—C3	−0.6 (4)	C10—C1—C11—N1	20.3 (4)
C11—C1—C2—C3	−178.2 (2)	C17—C12—C13—C14	0.2 (5)
O1—C2—C3—C4	−179.2 (3)	N1—C12—C13—C14	176.6 (3)
C1—C2—C3—C4	2.1 (4)	C12—C13—C14—C15	0.6 (5)
C2—C3—C4—C5	−1.9 (5)	C13—C14—C15—C16	−0.8 (6)
C3—C4—C5—C6	−179.5 (3)	C14—C15—C16—C17	0.3 (6)
C3—C4—C5—C10	0.3 (4)	C15—C16—C17—C12	0.5 (6)
C4—C5—C6—C7	−179.4 (3)	C15—C16—C17—C18	174.0 (5)
C10—C5—C6—C7	0.8 (5)	C13—C12—C17—C16	−0.7 (5)
C5—C6—C7—C8	−0.2 (6)	N1—C12—C17—C16	−177.2 (3)
C6—C7—C8—C9	−0.6 (6)	C13—C12—C17—C18	−174.2 (4)
C7—C8—C9—C10	0.7 (5)	N1—C12—C17—C18	9.3 (6)
C8—C9—C10—C5	0.0 (4)	C16—C17—C18—C19	67.1 (7)
C8—C9—C10—C1	178.2 (3)	C12—C17—C18—C19	−119.6 (5)
C6—C5—C10—C9	−0.7 (4)	C1—C11—N1—C12	−176.3 (3)
C4—C5—C10—C9	179.5 (3)	C13—C12—N1—C11	45.1 (4)
C6—C5—C10—C1	−179.0 (3)	C17—C12—N1—C11	−138.6 (3)
C4—C5—C10—C1	1.2 (4)	C1—C2—O1—C20	−165.1 (2)
C2—C1—C10—C9	−179.3 (3)	C3—C2—O1—C20	16.1 (4)
C11—C1—C10—C9	−1.9 (4)	C21—C20—O1—C2	170.8 (3)
C2—C1—C10—C5	−1.0 (4)		

### Hydrogen-bond geometry (Å, º)

**Table:**

*D*—H···*A*	*D*—H	H···*A*	*D*···*A*	*D*—H···*A*
C9—H9···N1	0.93	2.32	2.961 (4)	126
